# On the Neck as a Medical Region, and on Hidden Seizures

**Published:** 1849-11

**Authors:** Marshall Hall


### PATHOLOGY AND PRACTICE OF MEDICINE.

*On the Neck as a Medical Region, and on Hidden Seizures.* By Marshall Hall, M. D., F. R. S., &c.—I confess I was a little surprised in perusing the following note and confirmation of the views which have been laid before the reader. It is at once apt and very original, and denotes great talent in the writer.

59 Burton-crescent, March 23. 1849.

Dear Sir,—A few phenomena that have presented themselves, in a case in University College Hospital, during the last week or two, have so interested me, inasmuch as they bear upon your explanation of the second and third links in the chain of causes and effects, or symptoms of epilepsy, that I have taken the liberty of sending you an account of them.

A girl, nineteen years of age, was admitted (under Dr. Williams) for aphonia; and amongst other things in the treatment, she was ordered to have galvanism applied to the larynx, daily, by the electro-magnetic machine.

Whilst using this machine, I tried the effect upon the muscles of the neck, and observed, that when the wheel was turned slowly, and the superficial muscles were alternately contracted and relaxed, the color of the face was heightened, and was of a florid hue, and no unpleasant feelings (farther than those arising from the shocks) were experienced; but when the wheel was turned rapidly, with a less powerful current, and the muscles were maintained, during the rapidly intermitting action, in a state of almost permanent contraction, the face became of a deeper color, the lips and angles cf the mouth livid, the eyes suffused, and some feelings of confusion of thought, headach, and dimness of sight, alternating with flashing of light, were induced. The latter effects remained, after the cessation of the current, for a few minutes, and then disappeared.

I do not think that the treatment has, as yet, had any marked effect upon the aphonia; but the phenomena I have attempted to describe were interesting in their relation to the laws which you have established in your paper upon the 'Theory of Convulsive Diseases.'

I am, dear Sir, yours very respectfully, J. Russell Reynolds.

To Dr. Marshall Hall.

This interesting fact suggests a series of experiments on the lower animals, which I trust Mr. Reynolds will perform.

Not less remarkable are the facts contained in the following detail:—

On the 26th of February last I was called to see a patient, a butcher, aged forty, with Mr. Woolmer and Mr. Wall. Some months previously he had been observed to be absent in mind, and he had complained of severe pain of the right temple, especially on first awaking, and on the 25th of January, he had fallen down in his shop, taken with apoplexy. On this attack he was bled to twenty-four ounces, with material relief both to the apoplectic symptoms and the pain, and this being followed by calomel and other appropriate remedies, the patient was enabled to resume his occupation, going early to market, &c. Unfortunately, he was induced to resume a full diet, and to take tonics, and the symptoms returned; the countenance became flushed, the pupils dilated, and the pain returned with coma.

On February the 26th, when I first saw him, the eyes were suffused, the pupils sluggish; there was constant pain of the right temple, and extreme slowness in answering questions, the pulse 50; no hemiplegia. Venesection was ordered in the *erect* posture to incipient syncope, with aperient and light mercurial medicine.

Feb. 27th.—Thirty ounces of blood were taken before the slightest appearance of syncope was observed; the bowels had been freely moved.

The symptoms were all mitigated, but not removed; the pulse 60.

He was ordered to be cupped on the nucha to four ounces, the other remedies being continued. A spirit lotion was applied to the head, sinapisms to the nucha, fomentations to the feet.

28th.—There were still the pain of the right temple, suffused conjunctiva, and sluggish pupil; and the answers to questions were slower than yesterday, though the pulse had risen to 66.

The remedies were continued.

March 1st.—As yesterday. He grew worse towards night, with labored breathing, and died about ten P. M.

On a post-mortem examination, the external jugulars were observed to be tumid and cord-like; on removing the upper part of the skull, the veins were found excessively distended. The surface of the hemispheres were flattened. In the right anterior lobe of the brain, there were softening and slight discoloration, and a cavity communicating with the left ventricle, being distended with serum. The rest of the encephalon was healthy.

At this post-mortem, a brother of the deceased was present, himself a butcher, and he observed, in the most emphatic manner, "This appearance of the veins of the brain is precisely what we observe in the calf, if, after being struck with the pole-axe, we do not immediately proceed to stick the animal."

This is the singular fact to which I have alluded, and I took great pains to arrive at the precise truth in regard to it, both from the gentleman who first made the remark, and another person equally intelligent and well informed in the matter, to whom I submitted a series of questions. The facts, then, are these:

If a calf be struck with the pole-axe, and "stuck" *immediatety*, the brain is found pale-colored. But if it be struck with the axe, and not immediately stuck, the veins of the brain, the spinal marrow, and the neck, become enlarged, and gorged with blood. The interval required to produce this effect being about five minutes. The *eye* becomes suffused and red, and the tongue and internal parts of the mouth become livid. The tongue is sometimes thrust out and bitten. The breathing becomes very noisy and stertorous. There are strabismus and convulsive action of the muscles of the *face*, and of the *limbs*. The bladder and the rectum are sometimes evacuated. There is, in a word, every symptom of epilepsy!

It is impossble, I imagine, to read a detail more replete with interest, and I leave it for the present to the meditation of my reader. A volume might be written upon it. I proceed to another topic, adding merely that, first, through irritation of the nerves of the meninges, or, secondly, through that of the medulla oblongata, trachelismus and its train of consequences are induced.

What would be the precise distribution of an injecction thrown into the external jugular, the internal jugular, the vertebral, and the brachial, or axillary veins, singly and distinctly, respectively, in the human subject, as ascertained on a most careful dissection? What would be the precise effects of tying the two internal jugular veins, and the two vertebral veins, in distinct experiments in different animals? And what inferences might be legitimately drawn from such an inquiry? That the *accurate* pathology of phlebismus; or of interrupted circulation in the veins of the neck, is still to be ascertained, is indubitable. The effects of trachelismus observed in the apoplectic or epileptic seizure, of whatever degree, and in the case of strangulation, or of compression of the veins, however made, are too complicated to lead to any accurate and definite conclusion.

The anatomy, the physiology, and the pathology of the neck, present objects for new and important research. But the first step in the inquiry is taken when we first perceive and appreciate its value.

Two objects will principally occupy us in the present paper—the two principal causes of affection of the *circulation* in the encephalon and, in the enrhachidion, if I may use that term, are—1, *Inflammation*, and 2, *Congestion.*

Everything leads to the conclusion, that inflammation consists in interrupted flow of blood in the blood-channels situated intermediately between the minute branches of the arteries and the minute roots of the veins, and its effects. Congestion consists in the interrupted flow of blood along the veins. The former probably depends on an altered physical condition of the internal surface of the blood channels; the latter on compression of the venous trunks. There may be a point in which these two effects unite, in the latter case, when congestion may actually pass into inflammation. A diagram would best illustrate these two conditions.

The causes of inflammation act either immediately on the blood-channels, or through the medium of the *ganglionic system;* the causes of congestion, chiefly emotion and excitants of reflex action, act through the medium of the *spinal system.*

I have thus treated of the *theory* of these affections; I now add a most important remark, bearing upon *practice.*

It sometimes occurs that in the first instance, or in the midst of an apparent amendment, in diseases of the nervous system, a sudden retrograde change is experienced, for which no cause can be assigned.

I have reason to believe that in such cases some paroxysmal affection has occurred in the night, or in the day, unobserved

This fact, if established by careful observation, will raise the veil from many a mysterious event, and probably lighten the blame which the ignorant are so ready to throw upon the physician, who, forsooth, in such cases of difficulty and danger, is rendered responsible for every untoward event, when he has, in reality, no more power to control them than the astronomer has to control the course of the stars.

A passing emotion may induce trachelismus; this in its turn, phlebismus; and this, the condition of the nervous centres, which, remaining after the attack itself is over, is the cause—perhaps the hidden cause—of the origin, relapse, or augmented malady of the patient.

If this conjecture be just, how careful ought our researches and inquiries to be pursued on such occasions! The pillow, the tongue, the shirt, and the sheet, should be carefully examined; and suffusion of the eye, the pallor, flushing, or lividity of the countenance, should not be overlooked. Frequently the only evidence of an epileptic seizure having occurred during the night, is a wounded tongue or aching limbs.

But the seizure may be so slight as only to leave some such cerebral symptom, as confusion of intellect, or forgetfulness, or augmented excitability—events, the occurrence of which, however, it serves to explain.

These hidden seizures, occurring in the night, or in the absence of witnesses, as they are the cause of many cerebral and spinal symptoms, enable us to explain many occurrences which would remain enigmatical and mysterious. I cannot recommend the subject too earnestly for new investigation. Severe, attacks, whether apoplectic or epileptic, unfortunately present no difficulty in the diagnosis.

Perhaps I ought to apologize for the fragmentary character of these papers. I leave the *arrangement* of the subject for a later period of the investigation. In my next paper, I shall treat of a *cardiac* form of these affections: the medulla oblongata first, and then the heart, are affected with shock, and there is a *syncopal* paroxysm.—*London Lancet.*

